# Neurocognitive Impairment in Cardiovascular Disease Patients Taking Statins Versus Proprotein Convertase Subtilisin/Kexin Type 9 (PCSK9) Inhibitors: A Systematic Review

**DOI:** 10.7759/cureus.30942

**Published:** 2022-10-31

**Authors:** Rabia Shahid, Shaili S Naik, Shivana Ramphall, Swarnima Rijal, Vishakh Prakash, Heba Ekladios, Jiya Mulayamkuzhiyil Saju, Naishal Mandal, Nang I Kham, Pousette Hamid

**Affiliations:** 1 Internal Medicine, California Institute of Behavioral Neurosciences & Psychology, Fairfield, USA; 2 Internal Medicine, Surat Municipal Institute of Medical Education & Research (SMIMER), Surat, IND; 3 Internal Medicine, Government Medical College, Kozhikode, IND; 4 Psychiatry, California Institute of Behavioral Neurosciences & Psychology, Fairfield, USA; 5 Internal Medicine, Sree Narayana Institute of Medical Sciences, Ernakulam, IND; 6 General Surgery, Government Medical College, Thiruvananthapuram, IND; 7 Hospital Medicine, University of Medicine 1, Yangon, Yangon, MMR; 8 Neurology, California Institute of Behavioral Neurosciences & Psychology, Fairfield, USA

**Keywords:** pcsk9 inhibitors vs statins, subtilisin kexin neurocognitive effects, statins neurocognitive effects, pcsk9 neurocognitive effects, pcsk-9 inhibitors

## Abstract

Cardiovascular diseases (CVDs) are prevalent medical conditions affecting millions of people worldwide and are associated with significant morbidity and mortality. The main precursor of CVDs and the related events, such as hypertension and heart failure, is hyperlipidemia, most commonly an increase in low-density lipoproteins. Lipid-lowering drugs are cardinal in the treatment of CVDs. American College of Cardiology and American Heart Association have issued guidelines for lipid-lowering therapy, and statins are first-line medication. In the recent years, a new class of lipid-lowering agents called proprotein convertase subtilisin/kexin type 9 (PCSK9) inhibitors has been identified as the potential lipid-lowering therapy for the statin-resistant patient. In clinical trials and observational studies, PCSK9 inhibitors and statins are both associated with the development of neurocognitive dysfunction in the older population. This systematic review aims to inquire if there is significant neurocognitive dysfunction associated with statins and PCSK9 inhibitors and compare neurocognitive effects associated with statins with those of PCSK9 inhibitors.

## Introduction and background

Hyperlipidemia, with significantly elevated low-density lipoprotein cholesterol (LDL-C), is one of the major known modifiable risk factors for atherosclerosis that subsequently leads to a number of cardiovascular diseases (CVDs). Treatment of hyperlipidemia is imperative to prevent mortality and morbidity in millions of people suffering from CVDs worldwide. American Heart Association (AHA) released guidelines for the management of blood cholesterol [1]. According to the guidelines, statins are the first-line drugs for treating patients with hyperlipidemia.

Statins are hydroxymethylglutaryl-coenzyme A (HMG-CoA) reductase inhibitors. The liver regulates body lipids; LDL-C is taken up by hepatocytes at a 50% rate from the blood [2]. Statins work by inhibiting the enzyme HMG-CoA reductase, which converts HMG-CoA into mevalonic acid, a precursor to cholesterol.

Sterol regulatory element binding proteins (SREBPs) are cut out of the endoplasmic reticulum when HMG-CoA reductase is inhibited and translocate to the nucleus, where they boost the expression of the LDL receptor genes, which increases the LDL cholesterol uptake resulting in a decrease in intracellular cholesterol. Hepatic LDL receptors rise when hepatocyte cholesterol levels fall, which controls the decline in circulating LDL and its precursors like intermediate-density lipoproteins (IDLs) and very low density lipoproteins (VLDLs). After the administration of a single daily dose, the LDL cholesterol reduction with all statins is nonlinear and dose-dependent. Statins are the single most effective lipid-lowering drugs that decrease both morbidity and mortality associated with CVDs. The rupture of an unstable atherosclerotic lesion and the development of a thrombus is mostly the cause of coronary events. Statins inhibit the advancement of coronary atherosclerosis and cause its regression, as well as the development of new lesions thereby lowering the frequency of coronary events by a number of mechanisms [3]. LDL particles are more likely to enter the subendothelial compartment at lesion-prone artery sites when there is hypercholesterolemia (HC). Oxidized LDL and monocyte chemotactic protein-1 (MCP-1) operate as chemoattractants to promote monocyte accumulation and migration to the subendothelial region, where monocytes phenotypically change into macrophages. Oxygen free radicals alter LDL concurrently. Macrophage receptors that do not downregulate take up oxidatively damaged LDL to create lipid-rich foam cells. Foam cells transform into fatty streaks, which are the beginnings of atherosclerotic plaques. Statins have pleiotropic effects on a variety of atherosclerotic features associated with hypercholesterolemia, such as defective platelet coagulation, aberrant endothelial function, and factors influencing the thrombogenicity of plaques like inflammation and proliferation.

Statins are associated with a number of side effects, with the most commonly reported being myopathy ranging from myalgia to rhabdomyolysis [4]. Elevated hepatic transaminases generally occur in 0.5% to 2.0% of cases and are dose-dependent [5]. However, in recent years, cognitive impairment associated with statin use has gained much significance.

Among new lipid-lowering treatment modalities for statin-resistant patients, most important are proprotein convertase subtilisin/kexin type 9 (PCSK9) inhibitors. A number of PCSK9 inhibitors are being developed to block PCSK9 at various points in its life cycle as shown in Table 1. Three categories of inhibitory mechanisms exist: the inhibition of low-density lipoprotein receptor (LDLR) binding, inhibition of PCSK9 production, and inhibition of auto-catalytic processing [6]. The suppression of LDLR binding by PCSK9 enables a greater number of receptors to be recycled to the cell surface for further LDL-C elimination. PCSK9 synthesis inhibition involves preventing PCSK9 formation at the level of translation, silencing the PCSK9 gene. By interfering with PCSK9's auto-processing, the suppression of autocatalytic processing prevents cell maturation and secretion. The understanding of PCSK9 has led to the perception that a variety of techniques, including monoclonal antibodies, small interfering RNA (siRNA), vaccines, antisense oligonucleotides, small compounds, mimetic peptides, and adiponectin, can suppress its activity [7].

**Table 1:** PSCK9 inhibitors PSCK9: proprotein convertase subtilisin/kexin type 9; FDA: Food and Drug Administration; siRNA: small interfering ribonucleic acid

	Evolocumab	Alirocumab	Inclisiran
Brand name	Repatha	Praulent	Novartis
Type of compound	Monoclonal antibody	Monoclonal antibody	SiRNA
FDA phase	Approved	Approved	Approved

There are a number of side effects associated with PCSK9 inhibitors, like alirocumab and evolocumab, with the most common being nasopharyngitis, and injection site infection [8]. However, evidence from research conducted on evolocumab and alirocumab has revealed a greater frequency of cognitive side effects in patients taking PCSK9 inhibitors [9].

In this review, we aim to inquire about the neurocognitive dysfunction associated with statin and PCSK9 inhibitor use and assess if there is any correlation or causation between drug usage and the aforementioned outcome.

## Review

Methods

We searched through the articles in PubMed, Google Scholar, and clinical trials to find the relevant articles. After applying inclusion and exclusion criteria and quality assessment tools, we used the 20 articles to extract data and form results as shown in Table 2. We searched the database from May 11, 2022, to May 17, 2022, and used keywords like PCSK-9 inhibitors, PCSK9 neurocognitive effects, statins neurocognitive effects, subtilisin kexin neurocognitive effects, PCSK9 inhibitors vs statins.

**Table 2:** Keyword search through PubMed and Google Scholar PCSK9: proprotein convertase subtilisin/kexin type 9; MeSH: Medical Subject Headings

Keywords	Database	No. of articles	No. of articles after exclusion criteria
MeSH keywords	PubMed	87	16
PCSK-9 inhibitors	PubMed, Google Scholar	27002	18
PCSK9 neurocognitive effects	PubMed, Google Scholar	2063	17
Statins neurocognitive effects	PubMed, Google Scholar	8594	19
Neurocognitive dysfunction in patients with atherosclerotic cardiovascular diseases taking statins vs PCSK-9 inhibitors	Google Scholar	932	
PCSK9 inhibitors vs statins	Google Scholar	20400	
PCSK9 side effects	PubMed, Google Scholar	11224	229
Subtilisin kexin neurocognitive effects	PubMed, Google Scholar	1209	15
Statins and cognitive decline	PubMed, Google Scholar	3306	35

Inclusion/Exclusion Criteria

Randomized controlled trials (RCTs), case reports, observational studies, retrospective studies, and review articles were included in this review. Animal studies, articles not in English, and articles that were not free were not part of the review.

Results

**Figure 1:**
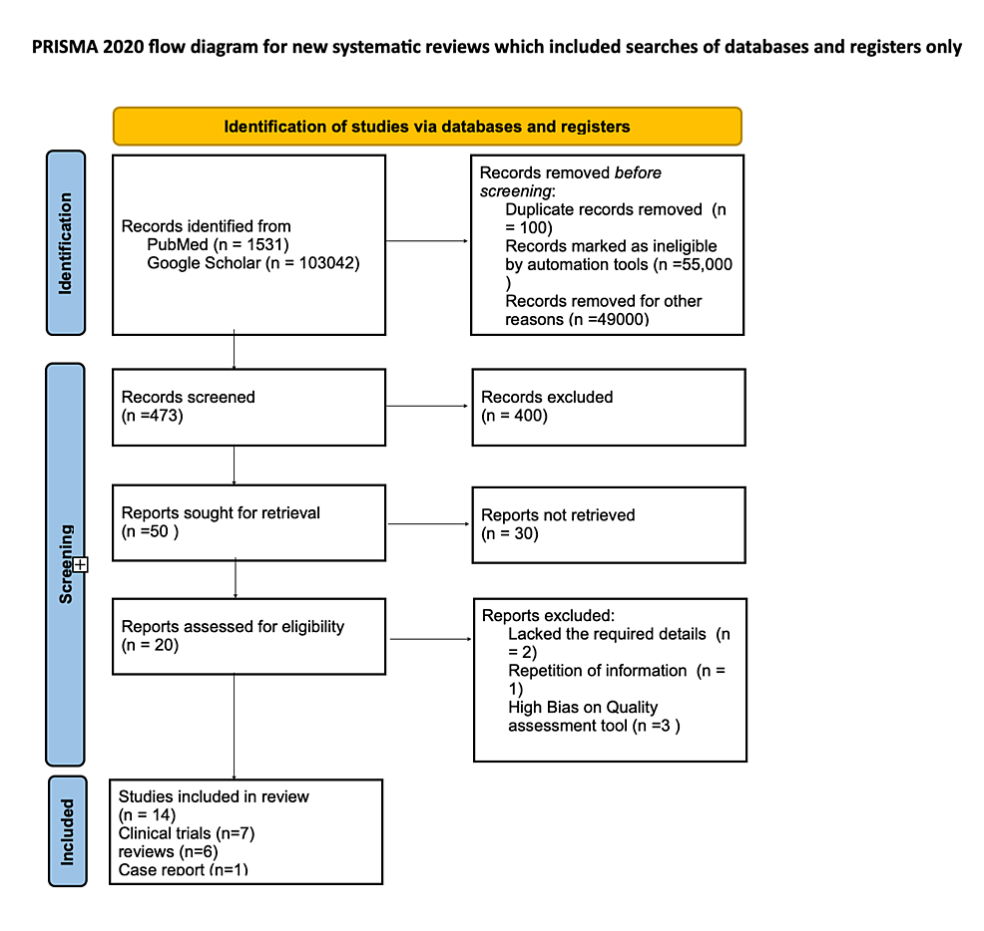
Flowchart of the literature review search per PRISMA 2020 guidelines PRISMA: Preferred Reporting Items for Systematic Reviews and Meta-analyses

We implemented Preferred Reporting Items for Systematic Reviews and Meta-analyses (PRISMA 2020) guidelines for systematic review as shown in Figure 1. A total of around 50 relevant articles were collected from PubMed and Google Scholar using Medical Subject Headings (MeSH) and regular keywords, respectively. The titles and abstracts of 50 articles were screened and only 20 articles were found relevant to this research topic; out of those, only 14 met the criteria using standardized quality assessment tools.

Characteristics of Studies Included

Clinical trials, narrative reviews and case reports were used for our systematic review, and their results pertaining to neurocognitive effects are mentioned in Table 3.

**Table 3:** Study characteristics OSLER-1: Open-Label Study of Long-Term Evaluation against LDL Cholesterol 1; ACC/AHA: American College of Cardiology/American Heart Association; PCSK9: proprotein convertase subtilisin/kexin type 9; FH: familial hypercholesterolemia; LDL-C: low-density lipoprotein cholesterol; EBBINGHAUS: Evaluating PCSK9 Binding Antibody Influence on Cognitive Health in High Cardiovascular Risk Subjects; ODYSSEY LONG TERM: Long-term Safety and Tolerability of Alirocumab in High Cardiovascular Risk Patients with Hypercholesterolemia Not Adequately Controlled with Their Lipid Modifying Therapy

S. no.	Author	Year	Study design	Study	Results
1.	Muldoon et al. [10]	2000	Investigational study	Cognitive assessment of patients treated with lovastatin vs placebo	Lovastatin was associated with a minor performance decline in attention and psychomotor speed neurophysiological tests
2.	Stone et al. [11]	2013	2013 ACC/AHA blood cholesterol guidelines	To create clinical practice guidelines for the treatment of hyperlipidemia	An assessment of the safety and efficacy of statins concluded that there Is no significant association of statins with cognitive impairment or dementia risk
3.	Rojas-Fernandez et al. [12]	2014	An assessment by the Statin Safety Task Force	To assess the effect of statin on cognition	They concluded that the neurocognitive effects of statins were not associated with cognitive dysfunction
4.	Ott et al. [13]	2015	Systematic review and meta-analysis	To assess the causal association between statin and cognitive dysfunction	They concluded that the strength of evidence is insufficient for the cognitive effects of statins
5.	Suraweera et al. [14]	2016	Case report	Report cognitive impairment associated with simvastatin	Simvastatin was associated with reversible cognitive dysfunction
6.	Schultz et al. [15]	2018	Narrative review	To assess cognition impairing and protective effects of statin	They concluded that statins were associated with reversible short-term memory loss and reduced risk of dementia
7.	Koren et al. [16]	2015	Open-label extension study	Open-label study, OSLER-1 extension study	Neurocognitive dysfunction 0.4% vs 0% in PCSK9 inhibitor vs statin group, respectively
8.	Sabatine et al. [17]	2015	Randomized controlled trial	OSLER-1, OSLER-2	The neurocognitive event associated with PCSK9 inhibitors <1%
9.	Robinson et al. [18]	2015	Randomized clinical trial	ODYSSEY LONG TERM	Neurocognitive dysfunction 2.9% vs 1.9% in PCSK9 inhibitor vs placebo group, respectively
10.	Kastelein et al. [19]	2015	Randomized clinical trial	ODYSSEY FH I and FH II	Neurocognitive effects for the PCSK9 group were 0.6% for FH I and 0% for FH II; for the placebo group, 1.2% vs 1.2% in FH I and II, respectively
11.	Ginsberg et al. [20]	2016	Randomized clinical trial	ODYSSEY HIGH FH	Neurocognitive dysfunction 2.8% vs 2.9% in PCSK9 inhibitor vs placebo group, respectively
12.	Khan et al. [21]	2017	Systematic review	Neurocognitive adverse effects of PCSK9 inhibitors	Their analysis showed PCSK9 inhibitor-associated neurocognitive effects statistically non-significant
13.	Giugliano et al. [22]	2020	Randomized clinical trial	EEBINGHAUS	LDL-C levels while receiving medication had no effect on cognitive function
14.	Zhao et al. [3]	2017	Systematic review and meta-analysis	Comparative efficacy and safety of lipid-lowering agents in patients with hypercholesterolemia	Neurocognitive effects are not associated with statins and PSCK9 inhibitors

Quality Assessment

We utilized Cochrane Collaboration’s tool for assessing the risk of bias for RCTs. Seven RCTs found to have a low risk of bias and of high quality were included in the systematic review as shown in Table 4.

**Table 4:** Cochrane Collaboration’s tool for assessing the risk of bias for randomized control trials

Studies	Selection bias - random sequence generation	Selection bias - allocation concealment	Performance bias - blinding of participants and personnel	Detection bias - blinding of outcome assessment	Attrition bias - incomplete outcome data	Reporting bias - selective reporting	Other bias	Overall risk of bias
Muldoon et al. [10]	Low	Low	Low	Unclear	Low	Low	Unclear	Low
Koren et al. [16]	Low	Low	High	High	Low	Low	Unclear	Unclear
Sabatine et al. [17]	Low	Low	Low	Low	Low	Low	Unclear	Low
Robinson et al. [18]	Low	Low	Low	Low	Low	Low	Unclear	Low
Kastelein et al. [19]	Low	Low	Low	Low	Low	Low	Unclear	Low
Ginsberg et al. [20]	Low	Low	Low	Low	Low	Low	Unclear	Low
Giugliano et al. [22]	Low	Low	Low	Low	Low	Low	Unclear	Low

Discussion

Neurocognitive dysfunction is characterized by the decline from a previously attained level of cognitive functioning, and includes delirium, mild cognitive impairment, and dementia. Diagnosis of neurocognitive dysfunction requires the assessment of all cognitive domains such as perceptual-motor function, executive function, complex attention, social cognition, learning and memory, and language [23]. The effect of lipid levels, especially LDL-C, on neurocognition has gained a lot of interest in the recent years.

Statins and Neurocognitive Dysfunction

The US Food and Drug Administration (FDA) in 2012 changed safety label instructions for lipid-lowering drugs like statins and added information about the possibility of relatively mild and reversible cognitive adverse effects (such as memory loss, confusion, etc.) [24]. They based these changes on reviewing the Adverse Event Reporting System (AERS) database including case reports, observational studies, and RCTs. The post-marketing adverse event reports mostly talked about people over 50 who had noticeable but vague memory loss or impairment that was curable if the statin medicine was stopped. The interval between the exposure to the statin and the event's commencement was diverse, ranging from one day to years. Analysis of a number of clinical trials showed the adverse effects of statin on cognition.

Muldoon et al. studied the effect of lovastatin on cognition [10]. In this double-blinded investigation, 209 otherwise healthy persons with a serum LDL cholesterol level of 160 mg/dL or greater were randomly assigned to receive lovastatin (20 mg) for six months or a placebo. At the beginning and end of the treatment period, the assessment of neuropsychological performance, which included general mental efficiency, psychomotor skills, attention, learning, and memory, and the assessment of psychological well-being for mood, hostility, anxiety, depression, hopelessness, anger, and social function were done. On a scale of standard deviation (SD), summarized effect sizes were assessed as z scores. A follow-up after six months showed improvement in all five cognitive function domains, namely, attention, psychomotor speed, mental flexibility, working memory, and memory recall in placebo-treated patients (all p<0.04), and the lovastatin-treated patients showed improvement in an only test of memory recall (p=0.03). This difference between the placebo- and lovastatin-treated groups was statistically significant for the test of attention (z score=0.18; 95% CI, 0.06-0.31; p=0.005) and for the test of psychomotor speed (z score=0.17; 95% CI, 0.05-0.28; p=0.004), indicating improvement in the placebo-treated patient group.

Stone et al. as a part of the American College of Cardiology/American Heart Association (ACC/AHA) Task Force created clinical practice guidelines for assessing cardiovascular risk, making lifestyle changes to reduce cardiovascular risk, manage blood cholesterol in adults, and manage overweight and obesity in adults [11]. They studied RCTs and systemic reviews and meta-analyses of RCTs; under the direction of the National Heart, Lungs, and Blood Institute (NHLBI), a formal peer review procedure that included 23 expert reviewers and representatives of government agencies was first completed. When the management of the guideline was transferred to the ACC/AHA, four expert reviewers were further asked to examine this paper. They assessed the safety and efficacy of statins and concluded that there is no significant association of statins with cognitive impairment or dementia risk.

Rojas-Fernandez et al. as a part of the Statin Cognitive Safety Task Force assessed the FDA updated guidelines for statins in 2014 [12]. Out of 14 RCTs, two showed cognitive impairment as a side effect of statin therapy. Muldoon et al. conducted two studies, one with lovastatin as described above and later with simvastatin to determine its effect on cognition in middle-aged men and women with hypercholesterolemia [10]. The study showed impairment in learning without memory deficit in performance testing for selected cognitive domains. There were baseline differences that acted as confounding factors and most results were equivocal. Also, the tests, which the authors referred to as "statin sensitive," also seemed to entail a variety of cognitive skills that are needed for and evaluated by the so-called "statin insensitive" effects. The clinical value of Muldoon et al.'s study is therefore unclear. To evaluate the effect of stopping statin and later re-starting it on cognitive function in diagnosed cases of Alzheimer's disease, Rojas-Fernandez et al. conducted a prospective 12-week open-label study that evaluated Mini-Mental State Examination (MMSE) and Consortium to establish a registry for Alzheimer's disease score. The MMSE score improved after stopping statin therapy (22.1 at baseline to 24 at six weeks after stopping statin therapy), and six weeks after re-starting statin therapy, the score was back to 22.1 (p=0.18). Despite variations favoring verbal memory improvement (p=0.04) with statin withdrawal and an improvement in language (p=0.01) with statin re-challenge, overall Consortium to Establish a Registry for Alzheimer's Disease scores remained similar. The lack of a control group, an open-label design, a small sample size, the length of the study, and uneven cognitive results were some of the study's limitations. They reviewed observational studies that included case-control, cohort studies, and narrative and systematic reviews that showed improvement in cognition among statin users versus nonstatin users; based on all the results, they concluded that the quality of evidence was low to moderate and strongly recommended that statins were not associated with cognitive dysfunction.

Ott et al. reviewed 25 RCTs with the primary objective of finding evidence of a causal association between statin therapy and cognitive impairment [13]. The secondary objectives included understanding whether those with cognitive impairments are particularly susceptible to any cognitive consequences and whether blood-brain barrier (BBB) penetrability differentiated between statins, regarding which were more likely to be linked with negative cognitive effects. Out of 25 RCTs, 18 trials included patients with normal baseline cognitive function, four RCTs included patients with Alzheimer's disease, and three RCTs considered patients with other cognitive impairments like traumatic brain injury or neurofibromatosis (NF) type 1. Three out of 18 trials with cognitively normal patients reported the development of cognitive adverse events (AEs). The JUPITER (or, Justification for the Use of Statins in Primary Prevention: an Intervention Trial Evaluating Rosuvastatin) trial comparing rosuvastatin vs. placebo showed cognitive adverse effects like dementia and confusion. The Heart Protection Study (HPS) comparing simvastatin versus placebo reported side effects like dementia and cognitive impairment. But cognitive side effects in both trials were statistically non-significant. One participant receiving a solution of 120 mg of atorvastatin had mild, temporary restlessness, exhilaration, and mental disorientation in a phase 1 dose-escalation study of atorvastatin vs. placebo done in 22 healthy young adults. Cognitive tests for global functioning, attention, executive functioning, memory, processing speed, and working memory domains were performed in all 16 RCTs that showed no statistical difference between patients taking statins vs placebo (standardized mean difference, or SMD=0.01; 95% CI, -0.01 to 0.03). BBB-penetrating statin subgroup analysis did not reveal any variations in effect size. The weighted mean difference for the Alzheimer's Disease Assessment Scale, Cognition (ADAS-cog) and MMSE instruments was higher in the statin arm compared to the control arm for trials with cognitively impaired patients (SMD=0.97; 95% CI, -2.21 to 4.16; p=0.403, and SMD=-0.67; 95% CI, -1.21 to -0.13; p=0.029, respectively) suggesting a trend towards benefit. However, the association was no longer significant after multiple comparisons were adjusted for or when analyses were repeated using net changes. In two small RCTs by the same research team, the impact of 10-day statin medication on the overall cognitive function of participants with acute traumatic brain injury was examined. Over a period of six months, one exhibited no statistically meaningful cognitive advantage, while the other did show a positive effect in four-month duration. In a short study of 62 children with NF type 1, expected to have a high prevalence of learning disabilities, statin therapy had no discernible effects.

Suraweera et al. reported two cases of simvastatin-associated cognitive decline [14]. One was of a 32-year-old Asian male diagnosed with bipolar disorder who complained of forgetfulness, short-term memory impairment, and impaired recall after he was prescribed simvastatin 20mg. The second case was of a 54-year-old Asian woman with treatment-resistant schizophrenia who developed memory impairment and difficulty in performing daily activities. Both patients developed symptoms two months after starting simvastatin and recovered within a period of three months and remained symptom free two years after the discontinuation of simvastatin.

Schultz et al. discussed both cognitive impairing and protective effects of statins in the aftermath of the FDA updates about statins [15]. They studied the results of statin trials with a focus on associated cognitive impairment. In the JUPITER trial, out of all participants (N=17,809) randomized between statin and placebo drug, only 12 and 9 patients, respectively, showed signs and symptoms of dementia and 18 patients from the statin group and four patients from the placebo group developed confusion. The major limitation of the study was that no standard tests for cognitive assessment were performed and the side effects mentioned were self-reported by patients. In HPS, researchers did assess cognition, but not at the baseline, and only at the last follow-up; in that trial, out of all participants (N=20,536) cognitive impairment was reported in 24% of the subjects. The extremely high finding of 24% cognitive impairment in each group may indicate that the technique employed to screen for cognitive impairment was not sensitive. In the Pravastatin in Elderly Individuals at Risk of Vascular Disease (PROSPER) trial, there was significant evidence of cognitive dysfunction with statin use; however, the blood-brain barrier has not been demonstrated to be permeable to pravastatin, and the results should not be generalized to other statins that do cross the BBB. On the evaluation of the AERS database, there were 2597 reports of cognitive impairment associated most commonly with the lipophilic statins when used in higher doses, e.g., simvastatin and atorvastatin. They concluded that the current research literature backs both reversible short-term cognitive impairment and decreased risk of dementia as potential effects of statins. However, the strength of the evidence is insufficient and has significant limitations specific to each study design.

Statin and Neurocognitive Dysfunction: Proposed Mechanism

Cholesterol is crucial for brain functioning; it is a structural component of the myelin sheath, and steroid hormones involved in signaling across the brain and peripheral tissue. It is essential for mitochondrial function, expression of neurotransmitter receptors, development of synapses, and transport of antioxidants like coenzyme Q10 [25]. The proposed mechanism is that the localized changes in the cholesterol level in the central nervous system (CNS) result in cognitive changes. Lipophilic statins (simvastatin, atorvastatin) especially at higher doses are most commonly associated with cognitive impairment, can cross the BBB, decrease cholesterol locally, and at higher doses can also passively diffuse across the blood-brain barrier. This mechanism may explain the reversible nature of the cognitive impairment because cholesterol levels return to normal after stopping the statin.

PSCK9 Inhibitors and Neurocognition

PCSK9 inhibitors such as monoclonal antibodies evolocumab (Repatha) and alirocumab (Praluent) were given the FDA approval to treat high cholesterol. The effectiveness of both of these in treating patients with atherosclerotic cardiovascular diseases (ASCVDs) was later demonstrated. PCSK9 inhibitors and their effect on neurocognition are documented in earlier trials of these drugs.

Koren et al. studied the results of the Open-Label Study of Long-Term Evaluation Against LDL Cholesterol 1 (OSLER-1) [16]. OSLER-1's primary objective was to ascertain whether evolocumab-induced LDL-C level reductions are sustained in various groups. The evaluation of adverse events, anti-drug antibodies, and factors influencing therapy discontinuation were secondary goals. A total of 1324 eligible patients were recruited and randomized in 2:1 to either group receiving evolocumab 420mg subcutaneously monthly with the standard of care (SOC) or only SOC. The baseline LDL level was 133mg/dL. At the follow-up at 52 weeks of open-label medication, median LDL-C levels were lowered by 61% (95% CI, 63% to 60%) from baseline in patients randomized to evolocumab plus SOC compared to 2% (95% CI, 5% to 0.2%) for those assigned to SOC alone (p<0.001). AEs occurred in 79.3% of patients using evolocumab plus SOC over the first year of evolocumab exposure compared to 74% of patients taking SOC alone during the 52-week SOC-controlled period. For neurocognitive events, the cumulative AE rates in the evolocumab plus SOC group were 0.4% versus 0% for SOC alone.

Sabatine et al. enrolled 4465 patients in two open-label, randomized trials OSLER-1 and OSLER-2 [17]. Patients were randomly assigned in a 2:1 ratio to receive either standard therapy alone or standard therapy plus evolocumab (140mg every two weeks or 420mg monthly). The patients were monitored for a median of 11.1 months, during which the cholesterol levels, among other parameters, were assessed. The two trials’ pooled data were analyzed. The main clinical hypothesis was that people with hypercholesterolemia would tolerate long-term treatment to evolocumab without adverse effects. The incidence of AEs served as the main endpoint for both trials. The percentage change in the LDL cholesterol level served as the secondary endpoint. The LDL level at the start of the trials was 120mg/dL; the outcome measure at 12 weeks showed LDL level reduction by 61% (95% CI, 59 to 63; p<0.001) in evolocumab versus SOC treatment. Adverse events related to evolocumab and standard therapy were 69.2% and 64.8%, respectively. Even though there were fewer neurocognitive adverse events (<1%), the evolocumab group reported them more frequently. It should be noted that the frequency of neurocognitive side effects did not seem to be connected to the LDL cholesterol levels during therapy. Delirium (including confusion), cognitive and attention disorders and disturbances, dementia, amnestic diseases, thinking and perception abnormalities, and mental impairment disorders were among the neurocognitive events.

Robinson et al. studied the results of long-term safety and tolerability of alirocumab versus placebo on top of lipid-modifying therapy in high cardiovascular risk patients with hypercholesterolemia (Long-Term Safety and Tolerability of Alirocumab in High Cardiovascular Risk Patients with Hypercholesterolemia Not Adequately Controlled With Their Lipid Modifying Therapy, or ODYSSEY LONG TERM) [18]. They randomized 2341 patients who were at a high risk of cardiovascular events in a 2:1 ratio to receive either alirocumab (150mg) or 1ml subcutaneous placebo injection every two weeks. The mean LDL of patients was 70mg/dL or more. Alirocumab caused a mean percentage change in estimated LDL cholesterol levels from baseline to week 24 of 61.0% versus 0.8% with placebo, showing a difference of 61.9 percentage points (p<0.001). The reports of side effects were the same in both study groups (for alirocumab, 81.0% vs 82.5%, for placebo). Neurocognitive adverse events occurred in 2.9% of patients taking alirocumab versus 1.9% in the placebo group. These included memory impairment, amnesia, and a state of confusion. They concluded that as no standard neurocognitive assessment tests were applied in testing the affected individuals, the validity of these side effects is questionable.

Kastelein et al. studied the efficacy and safety of alirocumab versus placebo on top of lipid-modifying therapy in patients with heterozygous familial hypercholesterolemia (FH) not adequately controlled with their lipid-modifying therapy (ODYSSEY FH I and ODYSSEY FH II) [19]. In these two trials, they randomized 486 for FH I and 249 for FH II in 2:1 to either receive alirocumab 75mg every two weeks (Q2W) or a placebo. The primary endpoint was to measure the change in LDL-C levels from baseline and at week 24. At week 24, mean LDL reduced from 144.7 to 71.3mg/dL in alirocumab-treated patient in FH I, and from 134.6 to 67.7mg/dL in FH II (p<0.0001). Treatment-emergent adverse events (TEAEs) were reported in 81.7% of the alirocumab group versus 79.1% of the placebo group in FH I, and in FH II, 74.1% and 81.5% in alirocumab and placebo groups, respectively. The occurrence of neurocognitive events in FH I was 0.6% and 1.2% for the alirocumab and placebo groups, respectively, and 0% and 1.2% for the alirocumab and placebo groups, respectively, in FH II.

Ginsberg et al. studied the efficacy and safety of alirocumab in patients with heterozygous familial hypercholesterolemia, with an LDL-C level of 160mg/dL or higher with a maximally tolerated statin (ODYSSEY HIGH FH) [20]. Every two weeks (Q2W) for 78 weeks, patients were assigned to receive 150mg of subcutaneous alirocumab or a placebo. The percentage change in LDL-C from baseline to week 24 was the primary outcome. The mean baseline LDL-C levels for the alirocumab group were at 196.3mg/dL while for the placebo group, they were at 201.0mg/dL. At week 24, alirocumab lowered absolute mean (SE) LDL-C levels by 90.8 (6.7) mg/dL compared to baseline, and the reductions persisted through week 78. In all, TEAEs were reported by 70.8% of patients receiving alirocumab versus 80.0% of patients receiving a placebo. Neurological incidents (placebo 2.9%, n=1, vs alirocumab 2.8%, n=2). Each group had one patient report a neurocognitive event: amnesia in the placebo group (2.9%) and disturbance in attention in the alirocumab group (1.4%).

Khan et al. studied the results of 11 studies to assess the safety with a focus on neurocognitive adverse events of PSCK9 inhibitors [21]. Out of the 11 studies, neurocognitive events were reported in eight studies. Adverse effects included delirium, cognitive and attention disorders, dementia and amnesia-like illness, deranged thinking and perception, and mental impairment disorders. A meta-analysis done by them showed no difference in neurocognitive event occurrence associated with the use of PSCK9 inhibitors (odds ratio, or OR, 1.29; 95% CI, 0.64-2.59; p=0.47). Based on these results, they concluded that although there is a possibility of neurocognitive adverse effects, there are limitations present in studies; for example, all events were self-reported by patients and thus lacked objective assessment of cognition, baseline cognitive assessment of patients was not performed and lastly, the percentage lowering of LDL-C was different in each population that may have some effect on cognitive outcomes.

Giugliano et al. recruited 1204 patients for evaluating PSCK9 binding antibody influence on cognitive health in high cardiovascular risk subjects (Evaluating PCSK9 Binding Antibody Influence on Cognitive Health in High Cardiovascular Risk Subjects, or EBBINGHAUS) [22]. This prospective evaluation of cognitive performance was conducted in patients with the past CVD treated with maximally tolerated statins who were participating in the Further Cardiovascular Outcomes Research With PCSK9 Inhibition in Subjects With Elevated Risk, or FOURIER trial (the randomized, double-blind, placebo-controlled cardiovascular outcome study of the PCSK9 evolocumab). Dementia, cognitive disability, or another serious mental or neurological conditions were important additional exclusion criteria for EBBINGHAUS. In this trial, objective tools for cognitive assessment, such as Cambridge Neuropsychological Test Automated Battery (CANTAB), were used. Comparing the spatial working memory (SWM) strategy index of executive function as evaluated by CANTAB in patients receiving evolocumab versus placebo was the primary endpoint. Comparisons of working memory, memory function, and psychomotor speed between evolocumab and placebo-receiving groups through various CANTAB sub-assessment tools were the secondary objective of the study. Cognitive assessment was performed at baseline, at week 24, once a year, and lastly, at the end of the trial. Participants in the EBBINGHAUS study also completed the Everyday Cognition (ECog) scale, a self-assessment of cognition, at the conclusion of the study. Researchers calculated that in order to have 97% power to detect non-inferiority, up to 1500 cognitively normal people would be needed, and they placed the non-inferiority border at 20% of the standard deviation. At baseline (17.8 for each patient) and at the conclusion of the trial (17.5 vs 17.6, respectively; p<0.0001 for non-inferiority), the results of the SWM strategy index were comparable between patients treated with evolocumab and placebo. Patients who were randomly assigned to receive either evolocumab or a placebo for medical treatment had similar results on the secondary objectives. Additionally, reports of cognitive side effects from both patients and researchers were consistent for both evolocumab and the placebo. The results of the investigation showed that LDL-C status while receiving medication had no effect on cognitive function; as a result, the cognitive function of patients with an LDL-C level of less than 25mg/dL was comparable to that of patients with higher plasma LDL-C levels.

PCSK9 Inhibitors and Neurocognitive Dysfunction: Proposed Mechanism

The exact mechanism that causes neurocognitive dysfunction in patients receiving PCSK9 inhibitors is not known. These monoclonal antibodies due to their larger size cannot cross the blood-brain barrier; therefore, the cognitive damage is due to the effect of lowered LDL-C in the body from their use. There are four proposed mechanisms: dysregulation of lipid and glucose metabolism, N-methyl-D-aspartate (NMDA) receptor modulation, alteration in neuronal cell membrane integrity, and predisposing risk factors like genetic variance [26].

Statins Versus PSCK9 Inhibitors: Neurocognitive Effects

Zhao et al. studied the lipid-lowering medication's comparative effectiveness and safety in people with hypercholesterolemia [3]. In terms of lowering all-cause and CV death, statins came out on top. The most successful cholesterol-lowering medications for raising lipid levels were PCSK9 inhibitors. In terms of neurocognitive effects, analysis of neurocognitive adverse events lacked clinical data. Statins (OR 0.99, 95% CI, 0.94-1.03) and PCSK9 inhibitors (OR 0.98, CI 0.94-1.03) did not significantly enhance the risk of major adverse events when compared to placebo. However, neither PCSK9 inhibitors (OR 1.26, CI 0.80-2.00) nor statins (OR 0.97, CI 0.51-1.86) were linked to an increase in neurocognitive events as compared to the placebo. Compared to PCSK9 inhibitors, statins ranked better in decreasing neurocognitive side effects.

Limitations

Small patient groups of the clinical studies included in this systematic review, to evaluate the neurocognitive effects of statins and PCSK9 inhibitors, may not have been representative of the general population. Also, studies included in this review were only those that were available as free full text that may have affected the results of this review.

## Conclusions

Statins and PCSK9 inhibitors both are imperative in the treatment of hyperlipidemia to reduce the risk of mortality and morbidity associated with CVDs. The neurocognitive dysfunction associated with these lipid-lowering agents includes reversible dementia, amnesia, delirium (including confusion), cognitive and attention disorders and disturbances, thinking and perception abnormalities, and mental impairment disorders. There was no causal relation found between low LDL-C levels and cognitive impairment among patients. The role of statins in both reversible cognitive impairment and treatment of cognitive dysfunction is conclusive. The association between neurocognitive dysfunction and lipid-lowering agents is not well established and there is a need for extensive long-term clinical trials to reach a conclusive decision. However, lipid-lowering agents' benefits far exceed the risks associated with them and should be part of the treatment regime of prospective patients. Healthcare workers have the responsibility of recognizing patient subgroups that are more vulnerable to the aforementioned side effects and performing cognitive assessment tests and informing patients about the possible side effects and managing them accordingly.
